# *In silico* Prediction and Exploration of Potential Bacteriocin Gene Clusters Within the Bacterial Genus *Geobacillus*

**DOI:** 10.3389/fmicb.2018.02116

**Published:** 2018-09-20

**Authors:** Kevin Egan, Des Field, R. Paul Ross, Paul D. Cotter, Colin Hill

**Affiliations:** ^1^School of Microbiology, University College, Cork, Ireland; ^2^APC Microbiome Institute, Cork, Cork, Ireland; ^3^Teagasc Food Research Centre, Moorepark, Fermoy, Co., Cork, Ireland

**Keywords:** bacteriocin, antimicrobial, bioinformatics, *in silico* screen, *Geobacillus*

## Abstract

The thermophilic, endospore-forming genus of *Geobacillus* has historically been associated with spoilage of canned food. However, in recent years it has become the subject of much attention due its biotechnological potential in areas such as enzyme and biofuel applications. One aspect of this genus that has not been fully explored or realized is its use as a source of novel forms of the ribosomally synthesized antimicrobial peptides known as bacteriocins. To date only two bacteriocins have been fully characterized within this genus, i.e., Geobacillin I and II, with only a small number of others partially characterized. Here we bioinformatically investigate the potential of this genus as a source of novel bacteriocins through the use of the *in silico* screening software BAGEL3, which scans publically available genomes for potential bacteriocin gene clusters. In this study we examined the association of bacteriocin gene presence with niche and phylogenetic position within the genus. We also identified a number of candidates from multiple bacteriocin classes which may be promising antimicrobial candidates when investigated *in vitro* in future studies.

## Introduction

The genus *Geobacillus* is composed of thermophillic, rod shaped, spore-forming, aerobic or facultative anaerobic bacteria. Their defining feature is their ability to grow at elevated temperatures of up to 80°C, with most isolates having growth temperature optima between 45° and 70°C (Nazina et al., 2001; Zeigler, 2014). Their sporulating nature makes their presence particularly challenging in food as they may survive intensive thermal processing methods and germinate when optimum conditions exist at a later period (Egan et al., 2016). In recent years this genus has attracted ever greater attention due an increased appreciation of its biotechnological potential, e.g., as sources of thermostable enzymes, as well as the biofuel and bioremediation industries (Cripps et al., 2009; Hussein et al., 2015; Kananavičiūtė and Citavičius, 2015; Studholme, 2015). One application of *Geobacillus* which has not yet been fully explored relates to their usefulness as a source of novel and highly potent antimicrobial peptides called bacteriocins.

Bacteriocins are ribosomally synthesized, narrow or broad-spectrum, antimicrobial peptides produced by bacteria. They can be broadly classified into three classes: class I post translationally modified, class II unmodified and class III <10 kDa in size (Arnison et al., 2013; Cotter et al., 2013). In past decades bacteriocins have been isolated primarily from lactic acid bacteria (LAB) due to their generally recognized as safe (GRAS) status which allows them to be used in food (Cotter et al., 2005). With the widespread use of *in silico* screening (Marsh et al., 2010; Azevedo et al., 2015; Walsh et al., 2015; Collins et al., 2017) and large culture based screening projects (Rea et al., 2010), bacteriocin candidates have been identified from alternative bacterial genera isolated from environmental, food or clinical samples. However, relatively few *Geobacillus*-associated bacteriocins have been identified to date (Pokusaeva et al., 2009; Garg et al., 2012; Özdemir and Biyik, 2012; Alkhalili et al., 2016), with very little genetic or structural information available with respect to these peptides. Geobacillin I and II represent the only two well characterized lantibiotic (class I) bacteriocins from this genus, with a large amount of information available with regard to antimicrobial spectrum, physiochemical characteristics and genetic determinants (Garg et al., 2012).

*In silico* screening of bacterial genomes for novel bacteriocins has become a staple element of bacteriocin discovery and characterisation over the past decade. Its widespread use and popularity has been driven by its ability to reduce time and cost relative to culture based bacteriocin screening studies. First generation *in silico* screening of bacterial genomes required the use of “driver genes” to predict potential new bacteriocin genes within genomes (Begley et al., 2009; Marsh et al., 2010). However, in recent years the bacteriocin prediction software BAGEL3 (van Heel et al., 2013) has become the tool of choice for *in silico* bacteriocin discovery. BAGEL3 searches bacterial genomes in DNA FASTA format using two different approaches to discover new bacteriocins, i.e., (1) detection of bacteriocin structural genes and (2) detecting other genes commonly associated with bacteriocin production. Those bacteriocins which are identified using both approaches are compared and filtered to remove duplicate candidates. Furthermore this software can be supplemented with traditional “driver gene” *in silico* screening or even with other programs such as antismash 3.0, which can detect other classes of antimicrobial peptides such as Non-Ribosomal PolyKetide (NRPK) antimicrobials (Weber et al., 2015).

This study set out to use BAGEL3 (van Heel et al., 2013) to perform an *in silico* screen of publically available *Geobacillus* genomes in an attempt to identify bacteriocin candidates for future *in vitro* experiments. The specific objectives were to (1) identify potential structural peptides within *Geobacillus* genomes; (2) investigate the possibility of a relationship between genome phylogenetic position and gene presence; and (3) examine any homology between structural peptide-encoding and surrounding genes with previously characterized bacteriocin gene clusters.

## Materials and methods

### Bacteriocin identification and visualization

Using the *in silico* bacteriocin prediction tool BAGEL3 (van Heel et al., 2013), genome sequences belonging to the genus *Geobacillus* (Table 1) were aquired and analyzed. Amino acid sequences of all 16 class III bacteriocins were aquired from Bactibase (Hammami et al., 2007) and aligned against the genomes as driver sequences using blastP (Altschul et al., 1990). Where necessary NisP (NCBI protein ID: AAA25200.1) and NisT (NCBI protein ID: AAA25191.1) driver sequences were used to seek and identify LanT and LanP-determinants in genome sequences. Those bacteriocin genes predicted were further visualized using Artemis genome visualization tool (Rutherford et al., 2000). Blastn and blastP (Altschul et al., 1990) were used to determine the % identities between putative peptides/genes and those accurately curated. Structural peptides were aligned using the Multiple Sequence Alignment (MSA) tool MUSCLE (Edgar, 2004) and then visualized using Jalview (Waterhouse et al., 2009). The previously generated MUSCLE peptide alignments were then input into the MEGA 7 software package (Kumar et al., 2016) for phylogenetic analysis. Using a neighbor-joining method, an unrooted phylogenetic tree was generated using a Jukes–Cantor method (Dukes and Cantor, 1969) and bootstrap replication values of 1,000 similarly to that by Zhang et al. (2015). In alignments where specific sequences contained no common sites, these were deleted. The resulting nexus tree files were exported to the interactive tree of life (itol) (Letunic and Bork, 2016) for graphical adjustment.

**Table 1:** List of *Geobacillus* genomes examined in this *in silico* screen.

**Number**	**Species**	**Strain ID**	**Accession no**.	**Source**	**Country**	**Sample type**	**Bacteriocin encoded?**
1	*G. galactosidius*	DSM18751	GCA_002217735.1	Compost	Italy	Environmental	Lantibiotic; LAPs
2	*G. icigianus*	G1w1	GCA_000750005.1	Hydrothermal samples	Russia	Environmental	No
3	*G. kaustophilus*	Et2/3	GCA_000948165.1	Geyser	Chile	Environmental	Circular; Sactipeptide
4	*G. kaustophilus*	Et7/4	GCA_000948285.1	Geyser	Chile	Environmental	Circular
5	*G. kaustophilus*	HTA426	GCA_000009785.1	Deep sea sediment	Marina trench	Environmental	Lantibiotic; circular
6	*G. litanicus*	N-3	GCA_002243605.1	High temp oilfield	Litunia	Environmental	Lantibiotic; Circular
7	*G*. sp.	Y4.1MC1	GCA_000166075.1	Hot Spring	USA	Environmental	LAPs; Class II
8	*G*. sp.	FJ8	GCA_000445995.2	Compost	Japan	Environmental	No
9	*G*. sp.	44B	GCA_002077755.1	Deep subsurface	USA	Environmental	Sactibiotic; LAPs
10	*G*. sp.	44C	GCA_002077865.1	Deep subsurface	USA	Environmental	Lantibiotic; Circular; LAPs
11	*G*. sp.	WCH70	GCA_000023385.1	Compost	USA	Environmental	Class II; LAPs
12	*G*. sp.	46C-IIa	GCA_002077765.1	Deep subsurface	USA	Environmental	No
13	*G*. sp.	47C-IIb	GCA_002077775.1	Deep subsurface	USA	Environmental	Sactibitoic
14	*G*. sp.	PA-3	GCA_001412125.1	Soil	Litunia	Environmental	Lantibitoic; Sactibitoic
15	*G*. sp.	12AMOR1	GCA_001028085.1	Deep sea hydrothermal vent	Unknown	Environmental	Sactibiotic
16	*G*. sp.	LEMMY01	GCA_002042905.1	Soil	Brazil	Environmental	Lantibitoic; Sactibiotic; Circular
17	*G*. sp.	1017	GCA_001908025.1	Oil water	China	Environmental	Lantibiotic
18	*G*. sp.	GHH01	GCA_000336445.1	Soil sample	Germany	Environmental	No
19	*G*. sp.	Y4.12MC61	GCA_000024705.1	Hot spring	USA	Environmental	Circular
20	*G*. sp.	Y4.12MC52	GCA_000174795.2	Hot spring	USA	Environmental	Circular
21	*G*. sp.	Sah69	GCA_001414205.1	Soil	Algeria	Environmental	Sactibitoic
22	*G*. sp.	JS12	GCA_001592395.1	Compost	South Korea	Environmental	Lantibiotic; Sactibiotic
23	*G*. sp.	T6	GCA_001025095.1	Hot water spring	Argentina	Environmental	Circular
24	*G*. sp.	BC02	GCA_001294475.1	Bore well isolate	Australia	Environmental	Circular; Sactibiotic
25	*G*. sp.	WSUCF1	GCA_000422025.1	Soil	USA	Environmental	No
26	*G*. sp.	FJAT-46040	GCA_002335725.1	Hot spring	China	Environmental	No
27	*G*. sp.	ZGt-1	GCA_001026865.1	Hot spring	Jordan	Environmental	Lantibiotic
28	*G*. sp.	A8	GCA_000447395.1	Deep mine	South africa	Environmental	No
29	*G*. sp.	CAMR5420	GCA_000691465.1	Unknown	Unknown	Environmental	No
30	*G. stearothermophilus*	10	GCA_001274575.1	Hot spring	USA	Environmental	Sactibiotic; Circular
31	*G. stearothermophilus*	22	GCA_000743495.1	Hot spring	Russia	Environmental	No
32	*G. stearothermophilus*	53	GCA_000749985.1	Hot Spring	Russia	Environmental	No
33	*G. stearothermophilus*	C1BS50MT1	GCA_001620045.1	Water sediment	Australia	Environmental	Circular
34	*G. subterraneus*	KCTC3922	GCA_001618685.1	Subsurface Oil field	China	Environmental	No
35	*G. subterraneus*	K	GCA_001632595.1	Oilfield	Russia	Environmental	No
36	*G. thermocatenulatus*	KCTC3921	GCA_002243665.1	Gas well isolate	USSR	Environmental	Lantibiotic; Circular
37	*G. thermocatenulatus*	BGSC93A1	GCA_002217655.1	Oilfield	Russia	Environmental	Lantibiotic; Circular
38	*G. thermocatenulatus*	SURF-48B	GCA_002077815.1	Deep subsurface	USA	Environmental	No
39	*G. thermodenitrificans*	NG80-2	GCA_000015745.1	Deep subsurface	China	Environmental	Geobacillin I; Geobacillin II
40	*G. thermodenitrificans*	T12	GCA_002119625.1	Compost	Neatherlands	Environmental	No
41	*G. thermoleovorans*	CCB US3 UF5	GCA_000236605.1	Hot spring	Malaysia	Environmental	Lantibiotic; Circular
42	*G. thermoleovorans*	FJAT-2391	GCA_001719205.1	Soil	China	Environmental	No
43	*G. thermoleovorans*	KCTC3570	GCA_001610955.1	Soil	USA	Environmental	No
44	*G. thermoleovorans*	N7	GCA_001707765.1	Hot spring	India	Environmental	Circular
45	*G. thermoleovorans*	B23	GCA_000474195.1	Deep oil reserve	Japan	Environmental	Lantibiotic
46	*G. uzenesis*	BGSC92A1	GCA_002217665.1	Oilfield	Russia	Environmental	No
47	*G*. sp.	B4113	GCA_001587475.1	Mushroom soup	Neatherlands	Food	LAPs; Circular
48	*G. kaustophilus*	NBRC102445	GCA_000739955.1	Pasteurized milk	Unknown	Food	Lantibiotic
49	*G. stearothermophilus*	A1	GCA_001183895.1	Milk powder facility	New Zealand	Food	Sactibiotic; Circular
50	*G. stearothermophilus*	B4114	GCA_001587395.1	Buttermilk power	Neatherlands	Food	Sactibiotic; Circular
51	*G. stearothermophilus*	D1	GCA_001183885.1	Milk powder facility	New Zealand	Food	Sactibiotic; Circular
52	*G. stearothermophilus*	P3	GCA_001183915.1	Milk powder facility	New Zealand	Food	Sactibiotic; Circular
53	*G. stearothermophilus*	DSM 458	GCA_002300135.1	Sugar beet juice	Austria	Food	Circular
54	*G. stearothermophilus*	GS27	GCA_001651555.1	Casein pipeline	Neatherlands	Food	Sactibiotic; Circular
55	*G. stearothermophilus*	ATCC 12980	GCA_001277805.1	Spoilled canned food	USA	Food	Sactibiotic; Circular
56	*G thermodenitrificans*	DSM 465	GCA_000496575.1	Sugar beet juice	Austria	Food	Lantibiotic
57	*G. thermodenitrificans*	KCTC3902	GCA_002072065.1	Sugar Beet juice	Austria	Food	Lantibiotic
58	*G. jurassicus*	NBRC107829	GCA_001544315.	Unknown	Unknown	Unknown	Sactibiotic
59	*G. kaustophilus*	GBlys	GCA_000415905.1	Unknown	Unknown	Unknown	Circular
60	*G*. sp.	G11MC16	GCA_000173035.1	Unknown	unknown	unknown	Lantibiotic
61	*G*. sp.	LC300	GCA_001191625.1	Bioreactor	USA	Unknown	Circular
62	*G*. sp.	C56-T3	GCA_000092445.1	Unknown	Unknown	Unknown	Circular
63	*G*. sp.	CAMR12739	GCA_000691445.1	Unknown	Iceland	unknown	Sactibitoic; Circular
64	*G*. sp	FW23	GCA_000617945.1	Oil well	India	unknown	Lantibiotic
65	*G. stearothermophilus*	ATCC7953	GCA_000705495.1	Unknown	Unknown	unknown	Circular
66	*G. subterraneus*	PSS2	GCA_000744755.1	Unknown	Unknown	unknown	Lantibiotic; Circular
67	*G. vulcani*	PSS1	GCA_000733845.1	Human Microbiome isolate	Japan	Human	Circular

*Also included is their accession numbers, location and type of bacteriocin predicted by BAGEL3*.

### Phylogenetic analysis of *Geobacillus* species

Where available, 16S sequences were acquired from genbank, however if no 16S sequence was available the *in silico* prediction tool RNAmmer (Lagesen et al., 2007) was used. The *B. cereus* ATCC14579 16S sequence was selected as a root for the final version of the tree. All 16S sequences were then collated and aligned as before using the MSA tool MUSCLE (Edgar, 2004). The resulting alignment output was then input into MEGA 7 (Kumar et al., 2016). Similar to Cihan et al. (2011), a neighbor-joining tree was generated using bootstrap values based on 1,000 replications and the resulting nexus tree file was then input into the itol software (Letunic and Bork, 2016) for final graphic adjustments. Where no common sites were found for specific peptides in the generation of the phylogenetic tree they were not included in the phylogenetic arrangement. The strains which had nether pre-determined or non-predictable 16S rRNA sequences were excluded from the overall study. The bacteriocin predictions by BAGEL3 were subsequently overlaid onto the phylogenetic tree using microsoft Powerpoint.

## Results

### Bacteriocin cluster distribution across the genus of *Geobacillus*

This study sets out to use an *in silico* approach to determine both the prevalence and diversity of bacteriocin gene clusters within the genus *Geobacillus*. Utilizing the genome sequences available in the public databases, 67 genomes (Table 1) representing 12 *Geobacillus* species, including *galactosidius, iciganius, jerrasicus, kaustophilus, liticanus, stearothermophilus, subterraneus, thermogalactosidius, thermoleovorans, thermocatenulatus, uziensis* and *vulcani* were analyzed. This screen resulted in the prediction of 88 bacteriocin gene clusters, of which 2 matched the previously characterized Geobacillin I and II (Garg et al., 2012) discovered in *Geobacillus thermodenitrificans* NG80-2. The other 86 clusters represented potentially novel bacteriocin candidates belonging to class I (modified) and class II (unmodified) bacteriocin families. When characterized class III bacteriocins were used as “driver” sequences and blasted against the entire *Geobacillus* genome database, no homologies were found. Furthermore no class III bacteriocins were predicted by BAGEL3.

**Figure 1:**
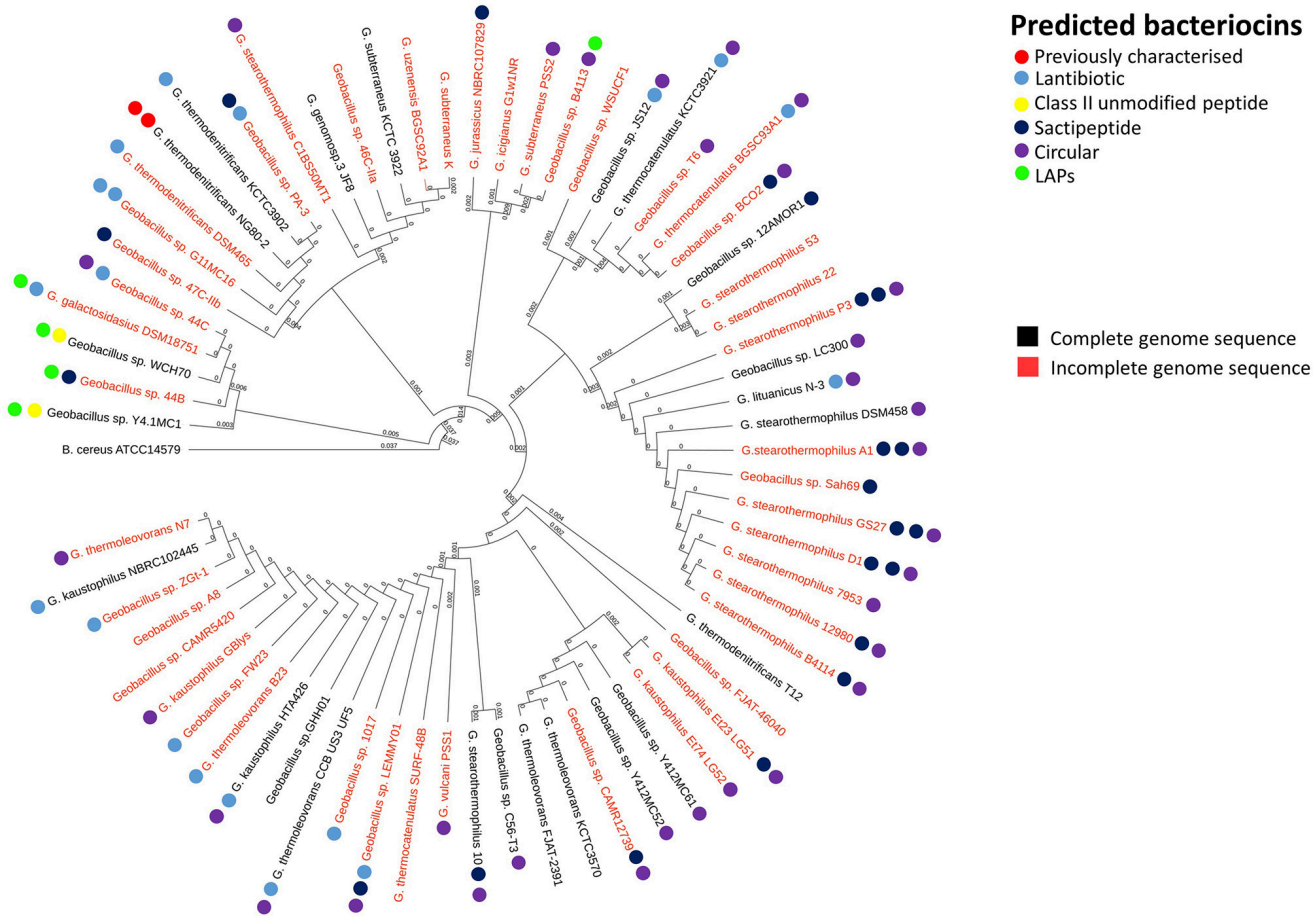
Phylogenetic arrangement of *Geobacillus* genomes investigated in this study. The BAGEL3 peptide predictions are overlaid in order to examine associations between bacteriocin gene presence and position within the *Geobacillus* phylogenetic arrangement.

In order to reveal associations between bacteriocin cluster gene presence within genomes and their phylogenetic position within the overall *Geobacillus* genus, we superimposed the BAGEL3 bacteriocin predictions onto a *Geobacillus* neighbor-joining phylogentic tree constructed from 16S rRNA sequences. Where possible, 16S rRNA sequences previously determined before whole genome sequencing (WGS) were used to construct the tree. However where no sequence was available, the 16S rRNA genes were predicted using *in silico* prediction software RNAmmer (Lagesen et al., 2007). Here we can see that bacteriocin clusters are both diverse and common across those genomes examined in this study (Figure 1). While lantibiotics and circular bacteriocin clusters are spread across the whole genus, Linear Azole-containing Peptides (LAPs), are associated with those strains for which a species has been designated but cluster closely with the species *G. galactosidius* and *G. thermodenitrificans*. A higher frequency of sactibiotics can also been seen within the species *G. stearothermophilus* but these are also present in other species. Furthermore, there are a number of strains included whose genomes have not been fully sequenced and therefore it is not possible to state definitively that alternative bacteriocin clusters are absent from these genomes other than those predicted in this screen.

Similarly to Walsh et al. (2015), the homology of predicted Potential Bacteriocin Gene Clusters (PBGCs) to existing genes and the arrangement of those genes was examined. Below we group PBGCs by bacteriocin class. These arrangements will display only those genes whose function is predicted to be involved in bacteriocin bioactivity and not those genes of unknown function that exist within these clusters.

### Class I bacteriocins

#### Lantibiotics

**Figure 2:**
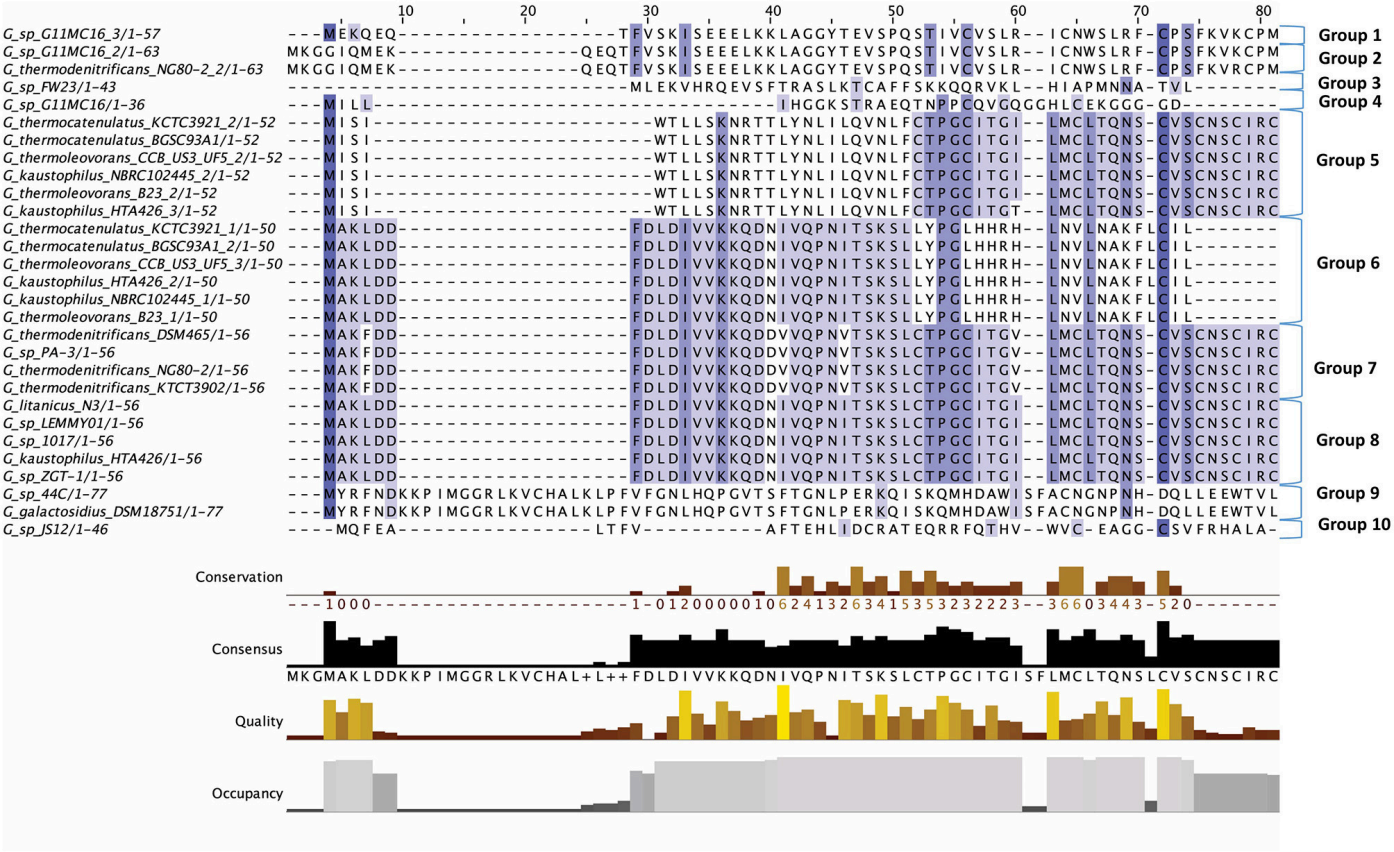
Multiple Sequence Alignment (MSA) of those lantibiotic peptides predicted. In some genomes multiple peptides were predicted within a single bacteriocin cluster and were therefore included as part of this alignment.

Twenty-nine putative lantibiotic gene clusters within 18 genomes were identified by BAGEL3 as part of this genome led bacteriocin screen (Figure 2). Lantibiotics belong to class I bacteriocins, which undergo significant post-translational modifications. These peptides are small and usually contain thioether internal bridges due to the interaction of dehydroalanine or dehydrobutyrine with intrapeptide cysteines, resulting in the formation of lanthionine or β-methyllanthionine residues. The structural gene (LanA) typically encodes a leader at the N-terminal of the prepeptide, which is transported across the cell membrane by LanT, then cleaved by LanP. The Post Translational Modification (PTM) enzyme LanB catalyzes the dehydration of amino acids, while LanC catalyzes thioether formation. The two component regulatory system genes, *lanR* and *lanK*, encode a response regulator and histidine kinase, respectively (Marsh et al., 2010; Draper et al., 2015; Field et al., 2015). While there are other PTM enzymes associated with lantibiotics they were not observed in this study so will not be described further, but they are discussed in greater detail elsewhere (McAuliffe et al., 2001).

The lantibiotics predicted in this study (Figure 2) were grouped according to their amino acid similarity. Grouping the predicted peptides in this way facilitates a comparison with characterized bacteriocins in Bactibase (Hammami et al., 2007). When aligned with the Bactibase bacteriocin peptide database the following highest homology hits was seen for each peptide group; Group 1: no hits; Group 2: 98% similarity to Geobacillin I; Group 3: no hits; Group 4: 12% similarity to LsbB; Group 5: 19% identity to nisin U; Group 6: 16% identity to nisin U; Group 7: 25% identity to nisin U; Group 8: 100% identity to Geobacillin I; Group 9: 5% identical to cinnamycin; Group 10: no hits. Furthermore, a phylogenetic analysis of those predicted peptides was carried out (Figure S1) resulting in the arrangement of 5 phylogroups. Phylogroups 1, 3, 4, and 5 were relatively homogenous showing little evolutionary distance between the group nodes. Phylogroup 2 however displayed a larger level of heterogenity with large evolutionary distances existing between the various nodes of the group.

**Figure 3:**
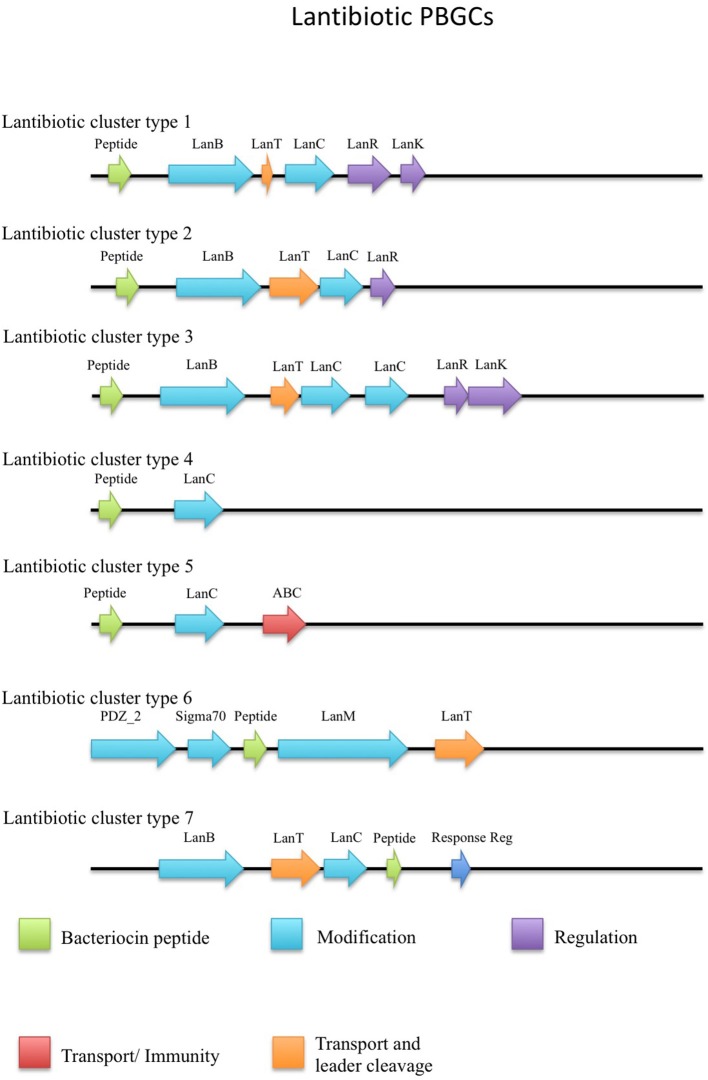
Lantibiotic cluster types predicted by BAGEL3.

The putative lantibiotics discovered consisted of 7 PBGCs (Figure 3) with some containing multiple peptide candidates per PBGC (Figure 2). These PBGCs were then typed according to their cluster structure so they could be easily compared with one another. The first cluster (lantibiotic cluster type 1) was contained within 9 genomes (*Geobacillus* sp. 1017, *G. thermocatenulatus* KTCT3921, *G. thermodenitrificans* KCTC3902, *Geobacillus* sp. PA-3, *Geobacillus* sp. Lemmy01, *G. kaustophilus* NBRC102445, *G. thermoleovorans* B23, *G. thermodenitrificans* DSM465, *G. thermocatenulatus* BGSC93A1). It consisted of genes predicted to encode a LanB, LanT, LanC, LanR and LanK consecutively and is similar to the Geobacillin I cluster with regard to its gene makeup. However, within this cluster structure, the predicted lantibiotic peptides were not completely homologous, showing differences in their amino acid composition (Figure 2). Additionally two adjacent lantibiotic peptides were predicted within this cluster type for the genomes: *G. thermocatenulatus* KTCT3921, *G. thermocatenulatus* BGSC 93A1 *G. thermoleovorans* B23 and *G. kaustophilus* NBRC102445. There were a number of exceptions to this general cluster structure: *G. thermoleovorans* CCB US3 UF5 and *G. litanicus* N3 lacked a LanK-determinant (lantibiotic cluster type 2), while *Geobacillus* sp. JS12 contained an extra LanC-encoding gene (lantibiotic cluster type 3). *G. thermoleovorans* CCB US3 UF5 encodes two peptides within this cluster type and they are located adjacent to each other. *Geobacillus* sp. 44C (Lantibiotic cluster type 4) encodes an identical peptide to *G. galactosidius* DSM18751 (lantibiotic type 5), but the PBGC of *G. galactosidius* DSM 18751 contains an additional ABC transporter after the LanC homolog. The genome for *Geobacillus* sp. G11MC16 is predicted to encode three LanA peptides. The first and second peptides are encoded within a distinct cluster from the third. These two peptides are within a cluster that also contains genes predicted to encode a PD2_2 homolog, sigma70, structural peptide, a LanM and LanT homolog (lantibiotic cluster type 6). The third putative peptide-encoding gene is not within an obvious PBGC, but is encoded 10kbs downstream of a region predicted to encode PTM enzymes SpaB-C, ABC transporter, LanC, LanR, and LanK. The peptide predicted to be encoded by *Geobacillus* sp. FW23 is within a cluster consisting of genes predicted to encode a LanB, LanT, LanC, structural peptide and response regulator (lantibiotic cluster type 7).

There were two putative LanAs encoded within the genome of *G. kaustophilus* HTA426. The gene corresponding to the first peptide was located upstream of three ABC transporter-determinants, while the gene corresponding to the second peptide was downstream of these three genes. There was a putative LanC and a further ABC transporter encoded approximately 10 kbs downstream from these predicted structural peptides which appear to exist within a neighboring gene cluster. However, no corresponding LanA-encoding gene was detected. The genome for *Geobacillus* sp. ZGt1 was predicted to encode one LanA that is situated upstream of two ABC transporter-encoding genes. However, the nearest putative LanB, ABC transporter and LanC determinants are located 10 kbs upstream of these genes. Finally NisP driver sequences were aligned against all genomes containing lantibiotics, however there were no definitive results which indicted the presence of these determinants.

#### Sactipeptides

**Figure 4:**
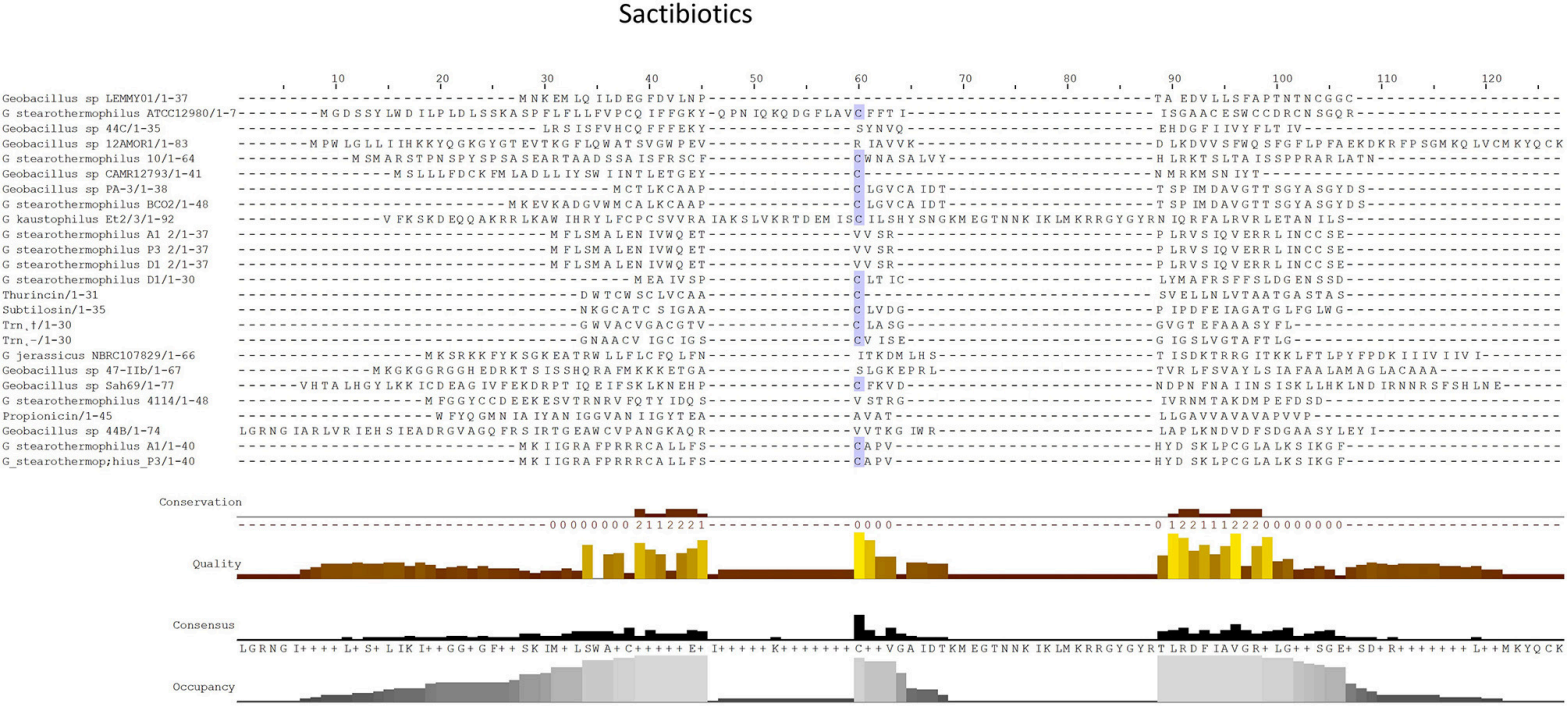
MSA alignment of Sactibiotic peptides predicted by BAGEL3 with characterized sactibiotic peptides.

Sactibiotics, like lantibiotics, are post-translationally modified and thus are a subclass of class I bacteriocins. These post-translational modifications take place in the form of intramolecular bridges of cysteine sulfur to α-carbon linkages (Mathur et al., 2015). 20 sactibiotics peptides were predicted within 17 *Geobacillus* genomes as part of this *in silico* screen (Figure 4). No conservation of amino acid residues was observed when these peptides were aligned with known sactibiotic structural peptides. Furthermore when these predicted peptides were aligned against the Bactibase bacteriocin peptide database (Hammami et al., 2007), no strong homologies with existing sactibiotics were found. Furthermore when a phylogenetic analysis of the predicted peptides (Figure S2) was carried out 3 phylogroups were observed. Phylogroup 1 contained the Trnα peptide while phylogroup 3 contained all other previously characterized sactibiotic peptides. Phylogroup 2 however did not contain any of the previously characterized sactibiotics.

**Figure 5:**
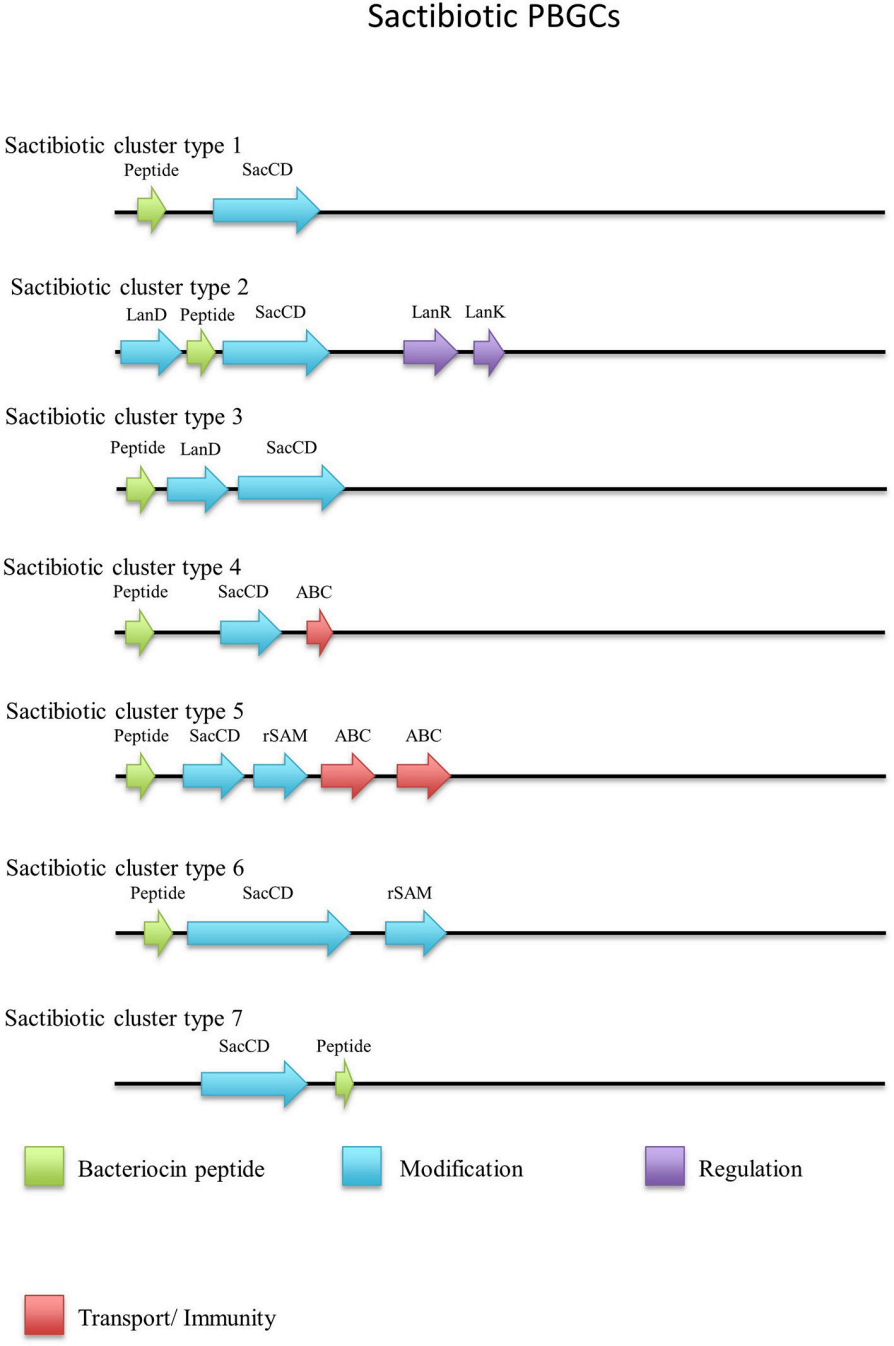
Sactibiotic cluster types predicted by BAGEL3.

When the sactibiotic biosynthetic gene clusters were further investigated, it was seen that 8 different types of predicted sactibiotic gene clusters were encoded within the *Geobacillus* genomes (Figure 5). The putative *G. stearothermophilus* A1, *Geobacillus* sp. GS27, *Geobacillus* sp. 47-IIb, *Geobacillus* sp. Sah69, *Geobacillus* sp. 44C, G. *stearothermophilus* ATCC 12980, *G. stearothermophilus* P3 and *Geobacillus* sp. BC02 SacA-determinants were all located upstream of a putative PTM enzyme SacCD-encoding gene (sactibiotic cluster type 1). *Geobacillus* sp. Lemmy 01 contained putative SacCD, LanK, LanR and LanD-encoding genes (sactibiotic cluster type 2). *G. jerrasicus* 107829 contained putative SacCD and LanD-determinants (cluster type 3). *Geobacillus* sp. CAMR12793 and *G. stearothermophilus* B4114 genomes encoded putative SacCD and an ABC transporter-determinants (Sactibiotic cluster type 4). *Geobacillus* sp. PA-3 contains putative SacCD, two ABC transporters and a Radical SAM enzyme-determinants (sactibiotic cluster type 5). The genomes for *Geobacillus* sp. 12AMOR1 and *G. kaustophilus* et2/3 contain putative SacCD and a radical sam enzyme-determinants (sactibiotic cluster type 6). *G. stearothermophilus* D1 and *G. stearotherophilus* A1 are predicted to encode peptides located downstream of a SacCD enzyme-determinant (cluster type 7).

The *G. stearothermophilus* 10 genome encoded a predicted structural peptide, radical SAM and two ABC transporters. While the structural peptide was encoded on the positive strand of the genome the two secondary enzymes were encoded on the negative strand and therefore are not part of the same operon but could however be part of this PBGC. A second putative sactibiotic gene cluster, predicted to be encoded within the *G. stearothermophilus* D1 genome, contains a structural peptide and SacCD enzyme-determinant, which are separated by 13 genes. The genome of *Geobacillus* sp. GS27 was predicted to encode a second sactibiotic peptide other than that predicted previously, however the SacCD-determinant driving this prediction was located on the opposite strand so is not encoded within the same operon but could still be part of the PBGC.

#### Linear azole containing peptides (LAPs)

**Figure 6:**
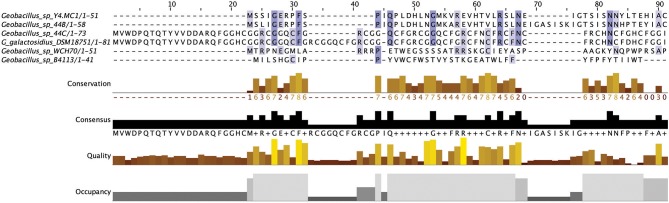
Linear Azole containing peptides predicted by BAGEL3.

Linear Azole containing Peptides (LAPs) are another subclass of class I bacteriocins that are distinguished by virtue of containing a variety of heterocyclic rings of thiazole and (methyl)oxazole. These are formed through an ATP-dependant cyclodehydration and further flavin mononucleotide-dependant dehydrogenation of the amino acid residues cysteine, serine and threonine. The most notable of the LAPs is streptolysin S, which is modified by the cyclodehydratase SagCD (Melby et al., 2011; Cox et al., 2015; Alvarez-Sieiro et al., 2016). Six putative LAPs were identified in six *Geobacillus* genomes (Figure 6), five of which were identified in those strains for which a species was not assigned. These peptides did not return any strong homologies to known LAPs or other bacteriocins when aligned against the bactibase bacteriocin database (Hammami et al., 2007). When a phylogenetic analysis of the predicted LAP peptides was performed (Figure S3), 3 phylogroups were observed, each consisting of two nodes.

**Figure 7:**
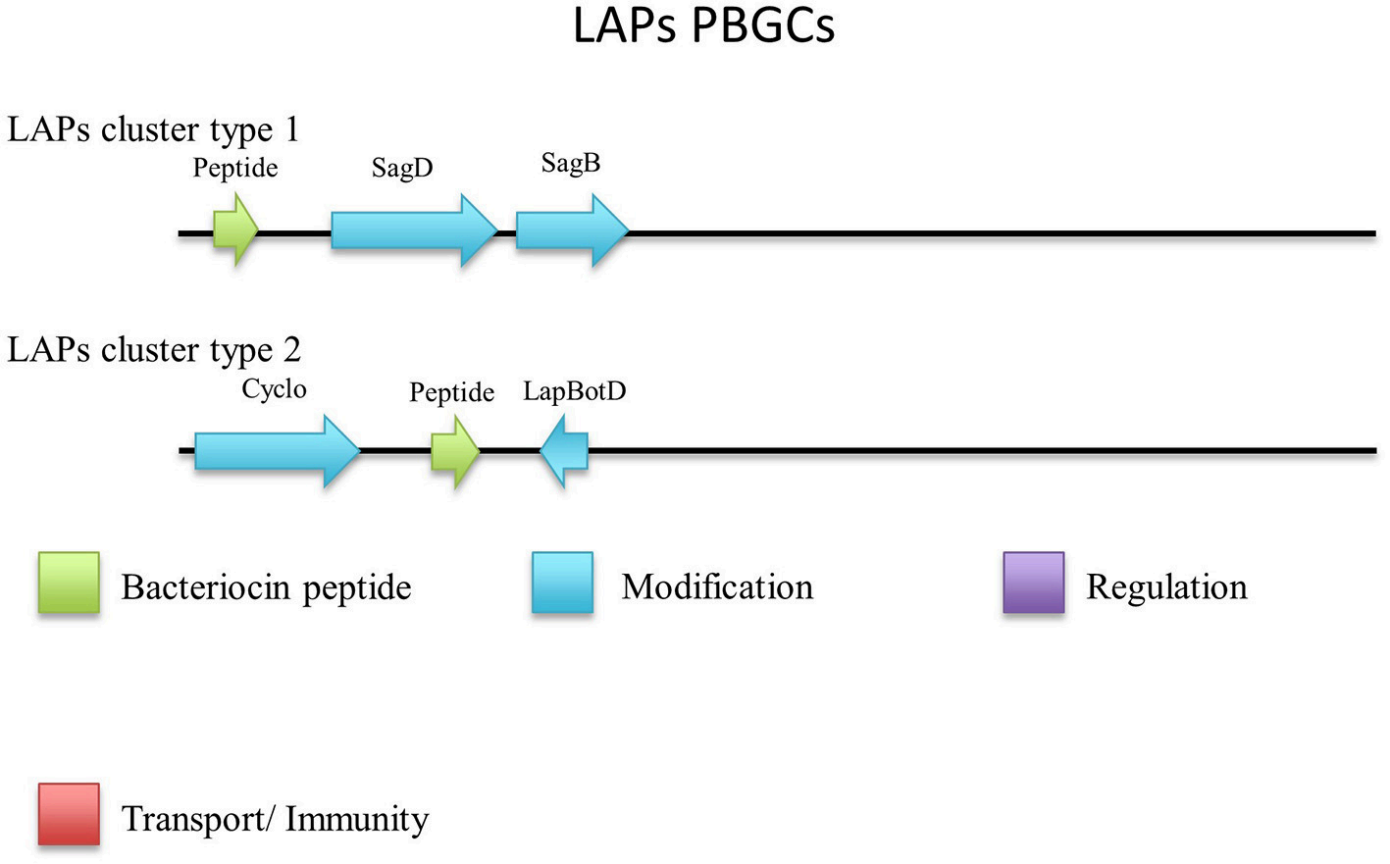
Linear Azole containing peptides (LAPs) cluster types predicted by BAGEL3.

Five out of six peptides (Figure 7) are contained within a gene cluster containing a structural peptide followed by a SagD-like and SagB-like determinants (LAP cluster type 1). For *Geobacillus* sp. B4113, the only gene which is predicted to be involved in the PTM of the associated peptide is a cyclodehydration enzyme-determinant upstream of the structural peptide (LAP cluster type 2). There is a LapBotD enzyme-derminant on the opposite strand which is close to the structural peptide, so while it is not part of the same operon it may still be part of this PBGC.

### Class II bacteriocins

#### Circular (a)

Circular bacteriocins belong to class IIc bacteriocins and are characterized primarily by the C to N terminal covalent linkage. They are known for their proteolytic, heat and pH resistance along with their size of 5.6–7.2 kDa, however to date only a handful have been characterized (Gabrielsen et al., 2014). Recently *in silico* software has been used to predict a new circular bacteriocin pumilarin (van Heel et al., 2017) and assisted in the characterisation of plantaricyclin from WGS data (Borrero et al., 2017).

**Figure 8:**
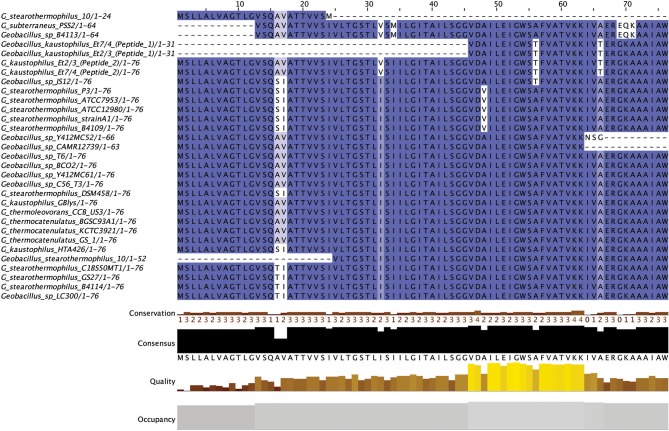
MSA of circular peptides predicted by BAGEL3.

**Figure 9:**
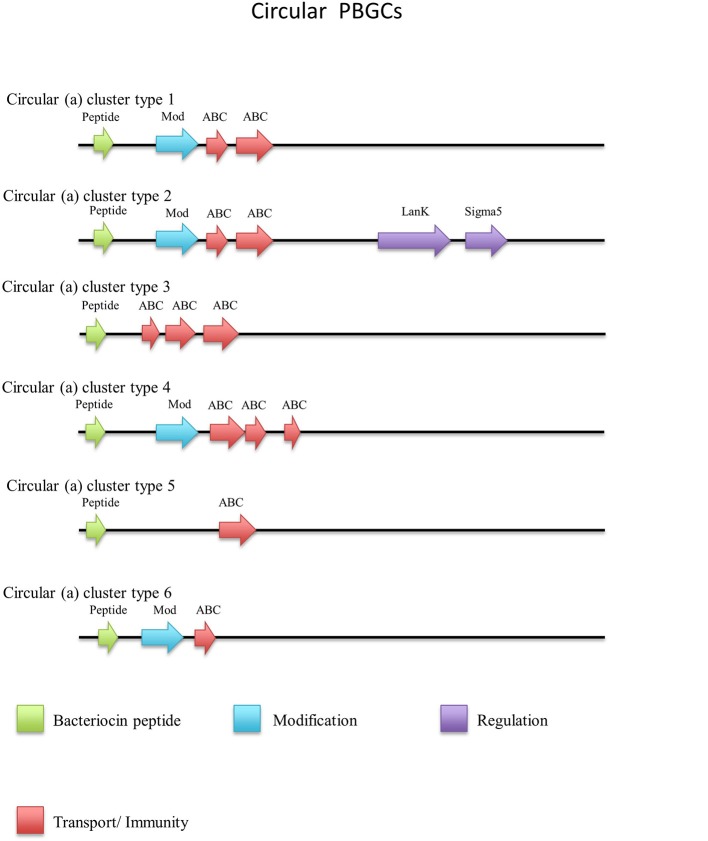
Circular (a) cluster types predicted by BAGEL3.

Thirty-one circular peptides were predicted within 29 genomes in this screen (Figure 8). These peptides displayed a weak homology (~30–40%) to known circular peptides when aligned against the bacteriocin database bactibase (Hammami et al., 2007). Five phylogroups were observed when a phylogenetic analysis of the peptides was performed (Figure S4). Three peptides from the strains *G. kaustophilus* Et7/4, *G. kaustophilus* Et2/3 and *G. stearothermophilus* 10 were not included in the phylogenetic tree due to the absence of common sites. While circular peptides have been predicted recently within the genomes of *Geobacillus* (van Heel et al., 2017), they have not been examined in terms of those genes which surround their structural peptide. For those circular structural peptides predicted within the genus, there are 6 general gene cluster structures (Figure 9). The genomes of *G. stearothermophilus* B4114, *G. stearothermophilus* GS27, *G. stearothermophilus* B4109*, G. stearothermophilus* 10, *G. stearothermophilus* ATCC12980, *G. stearothermophilus* A1, *G. stearothermophilus* ATCC7953, *G. stearothermophilus* P3, *Geobacillus* sp. 4113, *Geobacillus* sp. T6, *Geobacillus* sp. Y4.MC52, *G. thermocatenulatus* KTCT3921, *G. thermocatenulatus* BGSC93A1, *G. stearothermophilus* DSM458, *G. subterraneus* PSS2 and *Geobacillus* sp. Y412MC61 contain a cluster predicted to encode a structural peptide, a modification gene and two ABC transporter-determinants (Circular cluster type 1). The genomes of *Geobacillus* sp. JS12, *Geobacillus* sp. C56-T3, *Geobacillus* sp. LC300, *G. kaustophilus* Gbly and *G. thermoleovorans* CCB US3 UF5 contain a structural peptide gene, a modification gene, two ABC transporter genes and an additional 3 genes further downstream, putative LanK and Sigma5 determinants (circular cluster type 2). While it is unclear what role these gene products could play in the activity of the structural peptide, we do know that these genes are homologs of lantibiotic regulation machinery. The *Geobacillus* sp. BCO2 genome is predicted to encode a structural peptide and three ABC transporter-determinants (circular cluster type 3). *Geobacillus* sp. CAMR12739 is predicted to encode a structural peptide, a modification protein and three ABC transporter-determinants (circular cluster type 4). *G. kaustophilus* Et7/4 encodes a structural peptide and an ABC transporter-determinants (circular cluster type 5). The genome of *G. kaustophilus* Et7/4 encodes a modification and an ABC transporter-determinant following the structural peptide (circular cluster type 6).

#### Circular (b)

**Figure 10:**
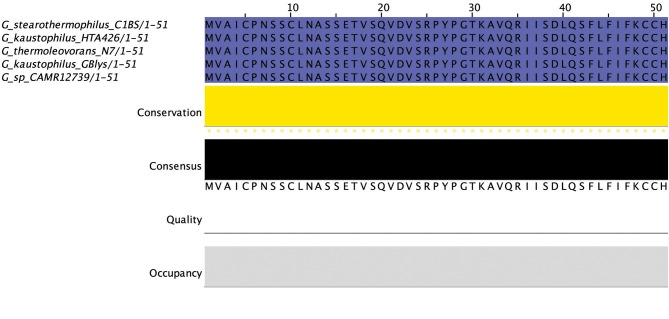
Circular peptides (b) predicted by BAGEL.

**Figure 11:**
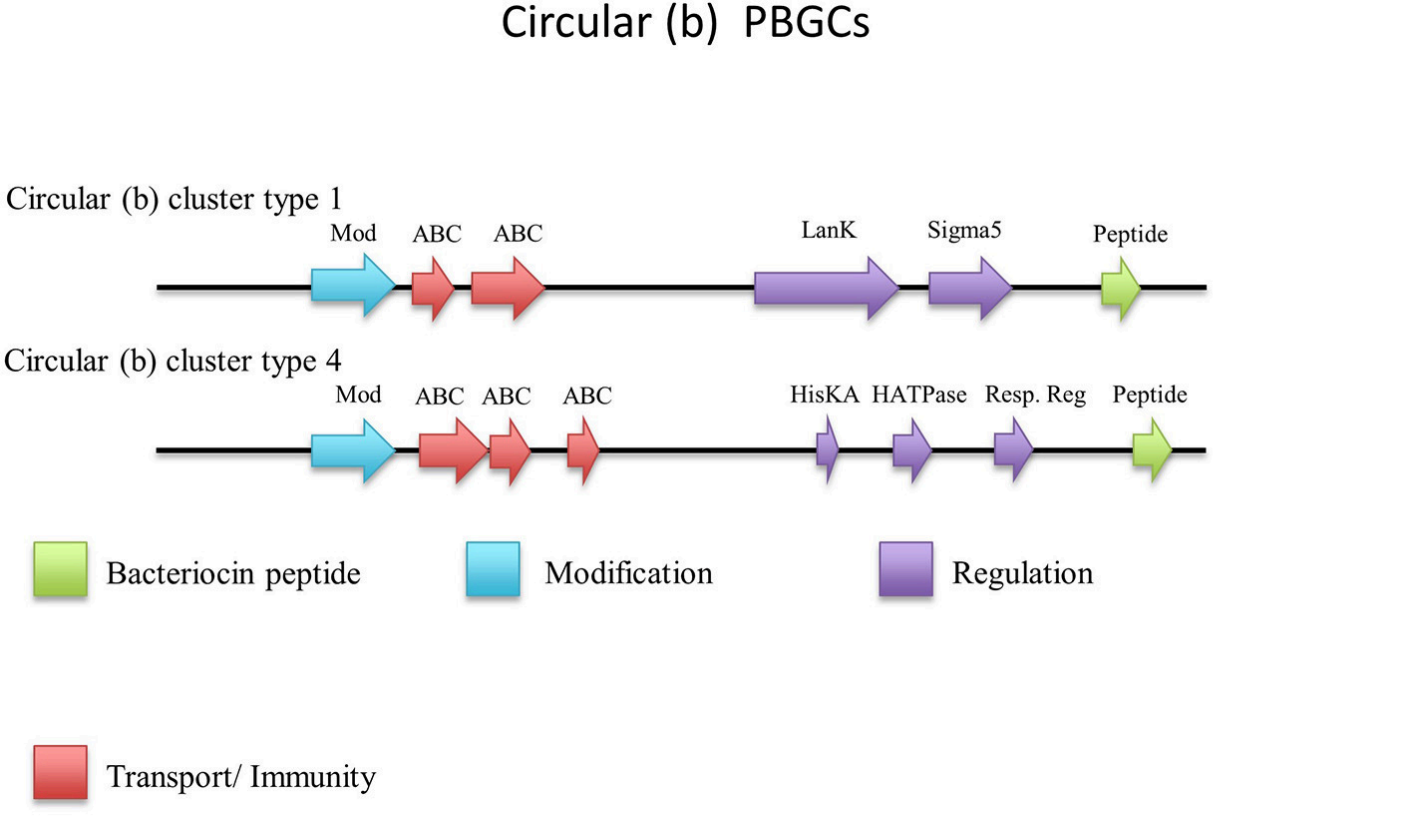
Circular (b) cluster types predicted by BAGEL.

There were an additional 5 identical circular peptides predicted (Figure 10) that had distinctly different amino acid sequences to the group a circular peptides described above and therefore were designated as a separate group. When aligned against the bacteriocin database bactibase these circular peptides displayed low homology of 17% to lacticin 481. Four of these predicted peptides were encoded within the genomes of *G. kaustophilus* HTA426, *G. thermoleovorans* N7, *G. kaustophilus* Gblys, *Geobacillus* sp. CAMR12739, *Geobacillus* sp. LC300. These circular peptides (Figure 11) were predicted to be encoded within the previously described type 2 circular PBGC (Figure 9). These structural genes were the last genes encoded within the cluster after the putative modification, 2 ABC transporters, LanK and Sigma 5 determinants (circular (b) cluster stype 1). It is unclear if these genes have any functional role within this cluster due to a lack of any strong amino acid similarity to known bacteriocins such as to circularin (Kawai et al., 2004) and enterocin AS-48 (Grande Burgos et al., 2014), however they could represent a family of potentially novel bacteriocins which may merit further *in vitro* testing.

The structural peptide predicted to be encoded by *Geobacillus* sp. CAMR 12793 is located approximately 10 kbs downstream of the previously described type 4 circular (a) cluster (b). It was accompanied by upstream putative histidine kinase and response regulator determinants (circular (b) cluster type 2) (Figure 11). As before it is unclear whether this peptide would be biologically active in this gene cluster so it may merit further *in vitro* experimentation.

#### Non circular class II bacteriocins

**Figure 12:**
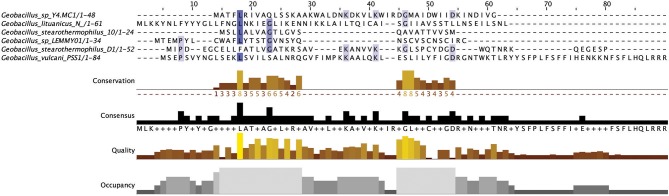
MSA of Class II bacteriocins predicted by BAGEL3.

**Figure 13:**
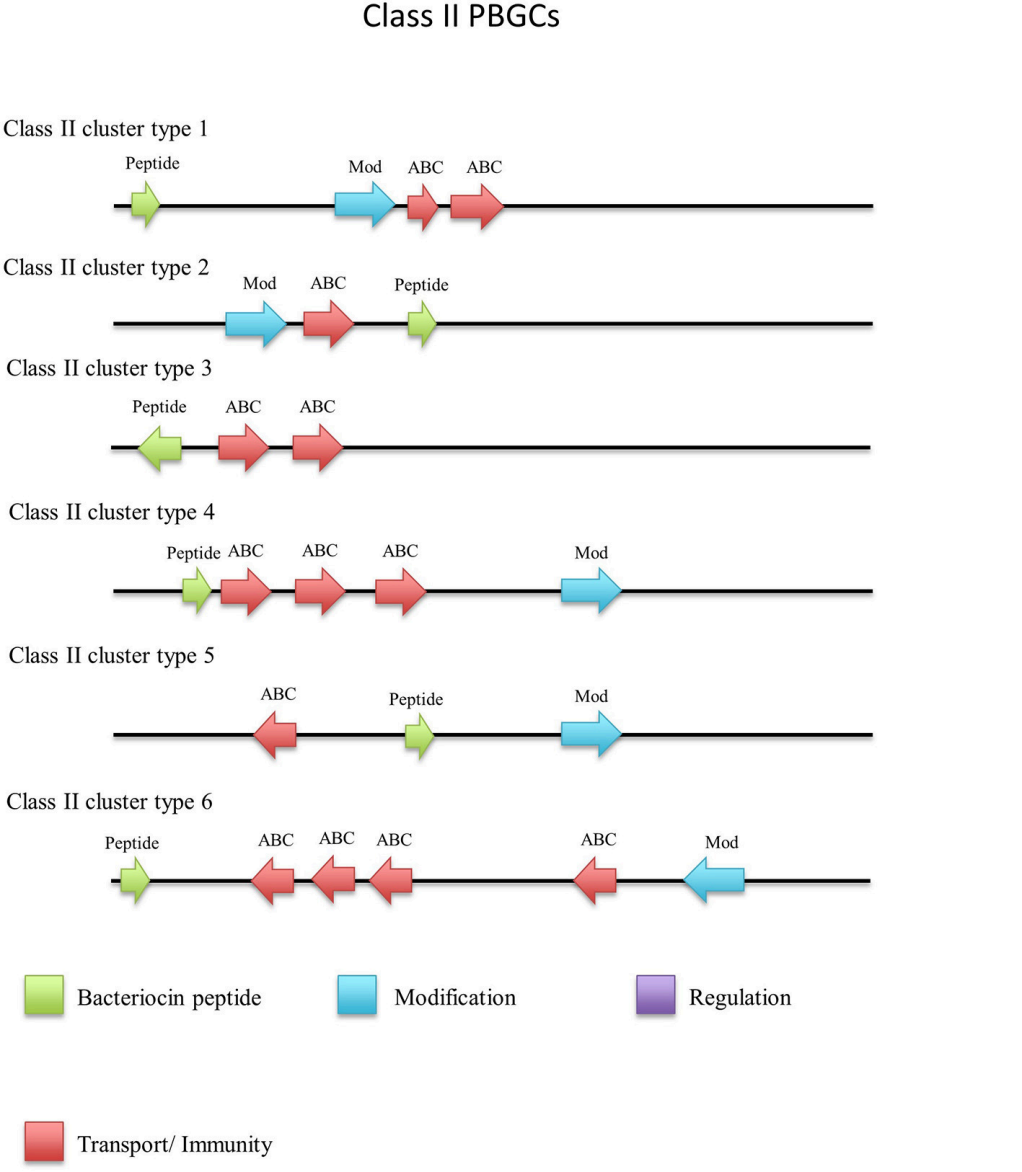
Class II cluster types predicted by BAGEL3.

There were a number of non-circular class II bacteriocins predicted by BAGEL3 (Figure 12), which are heterogenous with regard to both their predicted amino acid composition and those genes predicted to be involved in their bioactivity (Figure 13). When a phylogenetic analysis was performed, 3 phylogroups were observed for these predicted peptides (Figure S5). The predicted peptide encoded within the genome of *Geobacillus* sp. Y4MC1 structural peptide is 54% identical to Lacticin Z, however it is located on the opposite strand to its predicted ABC transporters so it is unclear whether they may have a role in its production (class II cluster type 3). The *G. stearothermophilus* D1 predicted cluster contained a circularisation enzyme and two ABC transporters, meanwhile the predicted structural peptide could be potentially novel as it displayed no homology to any known bacteriocins (class II cluster type 2). The *Geobacillus* sp. Lemmy 01 putative peptide did not display any homology to known bacteriocins and its prediction as a class II peptide was most likely based on the presence of a circularisation gene-determinant located 16 genes downstream of the structural peptide (class II cluster type 4). *G. stearothermophilus* 10 is predicted to produce a class II unmodified peptide (class II cluster type 1), which is encoded before the previously described circular (a) cluster type 1 (Figure 9). It is unclear if either or both peptides are bioactive. *G. litanicus* N3 is predicted to encode a bacteriocin which is two genes upstream of a circularisation gene-determinant, however it has no further transport or modification genes associated with it (class II cluster type 5). *G. vulcani* PSS1 encodes a class II peptide with no homology to existing bacteriocins and is situated on the opposite strand to four ABC transporter and modification gene-determinants (class II cluster type 6).

## Discussion

Bacteriocin prospecting has typically been a long and expensive process, based on trial and error in order to isolate bacteriocin producing bacteria and then optimize their growth conditions for bacteriocin production and protein purification. Further characterisation of these bacteriocins then typically required the use of trained personnel to carry out High Performance Liquid Chromatography (HPLC), mass spectrometry and other steps. Since the advent of *in silico* screening this process of bacteriocin discovery has been significantly reduced in terms of time and cost. Indeed this technology allows the bioinformatician to characterize to a high level putative bacteriocin candidates in terms of their amino acid content, physiochemical characteristics and surrounding genes which may be related to its function. Interestingly, it is these elements which had previously been the most laborious and expensive elements of bacteriocin discovery. This ability to identify candidates *in silico* ultimately removes a large portion of this trial and error process as so much is known about the bacteriocin once it is produced *in vitro*.

This *in silico* screen resulted in the identification of 7 lantibiotic, 7 sactibiotic, 2 LAPs, and 8 circular and 6 class II PBGCs which are potentially novel. The putative bacteriocins identified through this *in silico* screening approach will require further investigation through *in vitro* experimentation. However, it was possible to study the genes surrounding the structural peptide to more accurately predict that the bacteriocin cluster was indeed likely to be functional. Notably, in some cases those genes predicted by BAGEL3 were situated within annotated genes and could be determined to be pseudogenes. This study serves to therefore validate and critically assess BAGEL3 as a tool for bacteriocin discovery which could be advantageous for future improvements. When we consider the report that 30-99% of bacteria produce at least one bacteriocin Riley and Wertz (2002), it does seem likely that this may also be the case for *Geobacillus*, though a more complete picture will not become apparent until *in vitro* experiments are carried out to validate the findings of this study. Within the genomes examined here, only 23 of 67 were completely sequenced genomes. Where a genome only contains a partially sequenced bacteriocin cluster BAGEL3 will likely return a bacteriocin hit due to its dual detection method, distinguishing both structural peptides and associated bacteriocin genes. In order to fully explore the potential of *Geobacillus* as a reservoir of bacteriocin discovery, the generation of complete assembled genome sequences would be advantageous. A more conclusive picture of its potential will be revealed when the magnitude of genome sequences reaches that of *Lactobacillus*, which was examined recently *in silico* for its bacteriocinogenic potential (Collins et al., 2017). It could be expected that over the next number of years the amount of completely sequenced *Geobacillus* genomes will increase due to the wealth of data generated by way of the widespread use of metagenomic sequencing technologies and the ease/lower cost of WGS which is enabled by third generation sequencing technologies, such as PacBio (Rhoads and Au, 2015) and or Oxford Nanopore (Lu et al., 2016) instruments, that allow for *de novo* genome assembly. With this expected increase in genome sequence data, associations between niche and bacteriocin presence could be investigated in the future.

In the case of lantibiotic peptide predictions, LanT-determinants were not however identified always by BAGEL3 and in most cases LanT-determinant identification was made possible through the alignment of putative ABC transporter-determinants and NisT driver sequences, highlighting the importance of using a hybrid approach of BAGEL and driver sequence homology searching to peptide prediction. Furthermore, a LanK-determinant was absent from a number of lantibiotic gene clusters yet was found in circular PBGCs predicted in the same genomes. It is unclear what role (if any) these LanK-determinants play in these lantibiotic PBGCs. Another interesting observation which merits further investigation was the absence of LanP-determinants from the *Geobacillus* genomes as was seen in the study of the geobacillin I and II biosynthetic genes (Garg et al., 2012). This could be due to effects of incomplete genome sequencing or perhaps the absence of LanP-homologs for peptide leader cleavage as seen in geobacillin I and II. Issues surrounding absent bacteriocin gene-determinants have however been overcome in various studies through the use of heterologous expression systems and such technology will be important for future validation of the various *in-silico* screening studies that have taken place to date (Piper et al., 2011; van Heel et al., 2016; Mesa-Pereira et al., 2017).

A common method of bacteriocin molecular mass size determination involves the use of Native Sodium Dodecyl Polyacrylamide Gel Electrophoresis (SDS-PAGE), where the protein preparation is loaded onto an SDS gel and subjected to electrophoresis. It is then washed and overlaid with agar containing a sensitive indicator bacteria. A zone of inhibition surrounding a protein band provides an estimation of its molecular mass when compared to a molecular-weight size marker or ladder. While we have seen this method used to estimate the molecular mass of a bacteriocin produced by *Geobacillus* sp. ZGt-1 of 15–20 kDa, no such class III bacteriocin was predicted within this genome in our *in silico* screen (Alkhalili et al., 2016). This may indicate the presence of a potentially highly novel class III bacteriocin within *Geobacillus* sp. ZGt-1 given the lack of homology to any known class III peptide, the presence of an uncommon gene cluster not identified in this study or the presence of another type of peptide antimicrobial other than a bacteriocin. Toebicin 218 is produced by *G. stearothermophilus* DSM22 with a molecular mass of 5.5 kDa (Özdemir and Biyik, 2012) and it is interesting to note that no bacteriocin was detected within this genome in the current study. Pokusaeva et al. (2009) used this method to estimate the size of bacteriocins produced by various *G. stearothermophilus* at 6.8, 5.6, 7.1, and 7.2 kDa. However, the genomes of these strains have not been sequenced and therefore the identity of potential bacteriocin candidates cannot be determined through bioinformatics. This is also the case for *G. toebii* HBB-247, that has been shown to produce a bacteriocin with an estimated mass of 38 kDa (Başbülbül Özdemir and Biyik, 2012; Özdemir and Biyik, 2012). There are a number of other bacteriocins of undetermined mass which have been characterized within *Geobacillus* prior to modern sequencing or mass spectrometry methods (Shafia, 1966; Yule and Barridge, 1976; Sharp et al., 1979; Fikes et al., 1983). Indeed, it is notable that there is a significant lack of mass spectrometry data for all *Geobacillus*-associated bacteriocins other than the lantibiotics Geobacillin I and II discovered within *G. thermodenitrificans* NG80-2.

While *Geobacillus* appears to represent a potential reservoir for novel bacteriocin discovery, its route to commercial application in food or medicine remains unclear. The nature of *Geobacillus* when in the form of a thermally resistant spore makes it difficult to remove once introduced into an processing environment (Egan et al., 2016). Furthermore, the associated high temperature growth requirements would translate to high processing and energy costs. Typically its direct addition to food, albeit a GRAS bacterial genus, is not applicable due to its history as a bacterial spoilage agent. Despite this, *Geobacillus* do already have applications in the biotechnology industry in a number of ways (such as biofuel and chemical production), so perhaps it is within this niche where bacteriocins produced by *Geobacillus* could be of commercial relevance. Additionally, these bacteria could serve as a platform for research into protein thermostability and as a source of not only heat stable bacteriocins but also post translational modification enzymes. Finally, with the oncoming antimicrobial resistance (AMR) crisis, humankind is looking outside of the traditional antimicrobial candidate reservoirs and increased investment in other classes of antimicrobials such as defensins (Oppedijk et al., 2015) are visibly apparent. Given the abundance of potentially novel bacteriocins identified by this study, *Geobacillus* spp. could yet develop their full potential as a source of new peptide structures with enhanced functionality.

## Author contributions

KE drafted the manuscript. KE and CH conceived the manuscript. DF, RR, PC, and CH revised and approved the final manuscript.

### Conflict of interest statement

The authors declare that the research was conducted in the absence of any commercial or financial relationships that could be construed as a potential conflict of interest. The reviewer GG and handling Editor declared their shared affiliation

## Supplementary material

Figure S1 Phylogenetic arrangement of predicted lantibiotics.

Figure S2 Phylogenetic arrangement of predicted sactibiotics.

Figure S3 Phylogenetic arrangement of predicted LAPs.

Figure S4 Phylogenetic arrangment of predicted circular bacteriocins.

Figure S5 Phylogenetic arrangement of predicted class II bacteriocins.

## Abbreviations

LAPs: Linear Azole-containing Peptides
PTM: Post Translational Modification
PBGCs: Potential Bacteriocin Gene Clusters
WGS: Whole Genome Sequencing.
