# Supplementary figures and images for: In silico Prediction and Exploration of Potential Bacteriocin Gene Clusters Within the Bacterial Genus Geobacillus
Source: Front Microbiol. 2018 Sep 20;9:2116. doi: 10.3389/fmicb.2018.02116 (PMC6160750; doi:10.3389/fmicb.2018.02116)

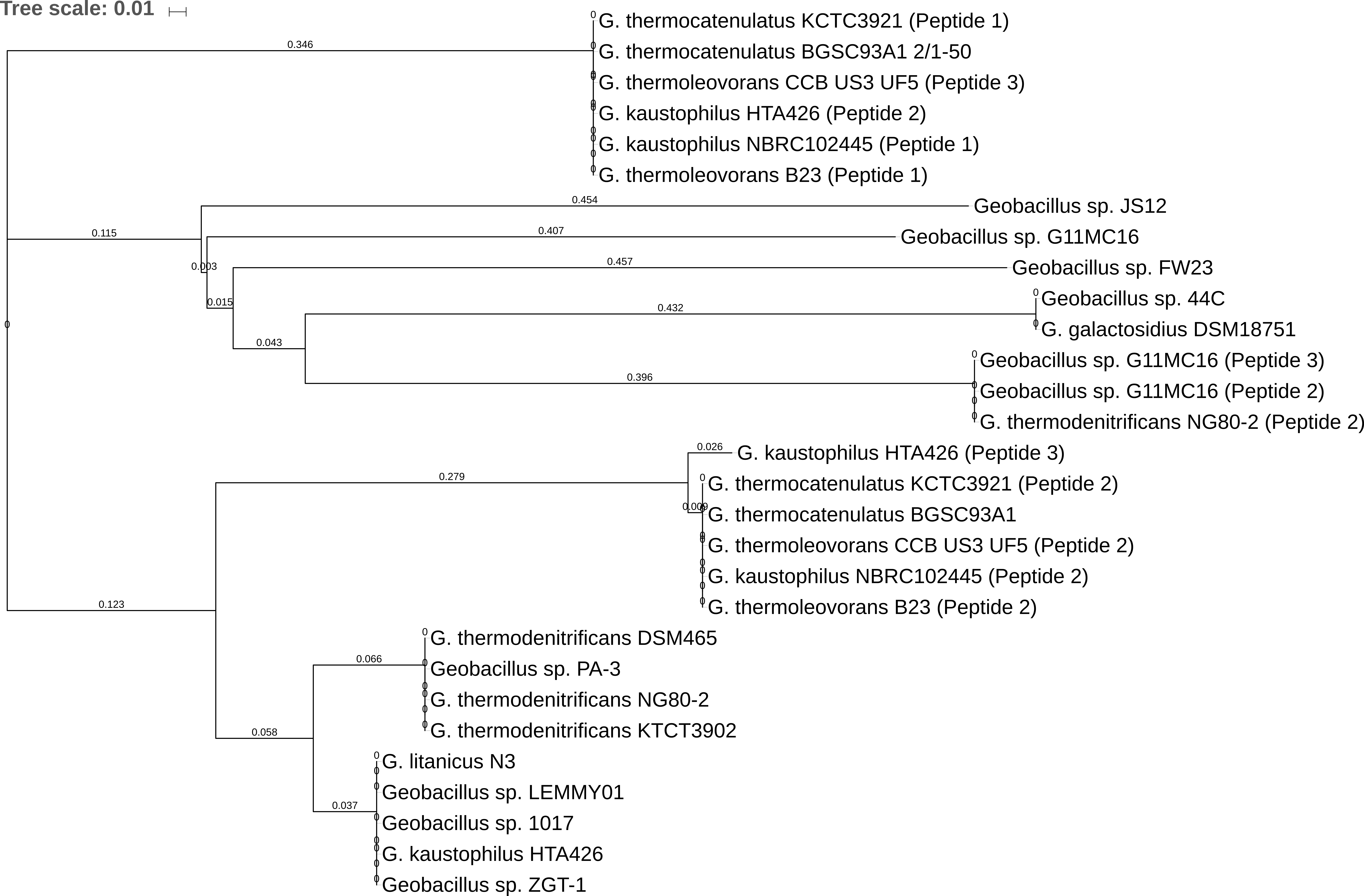

Supplement: Figure S1 — Phylogenetic arrangement of predicted lantibiotics. [file Image_1.JPEG]

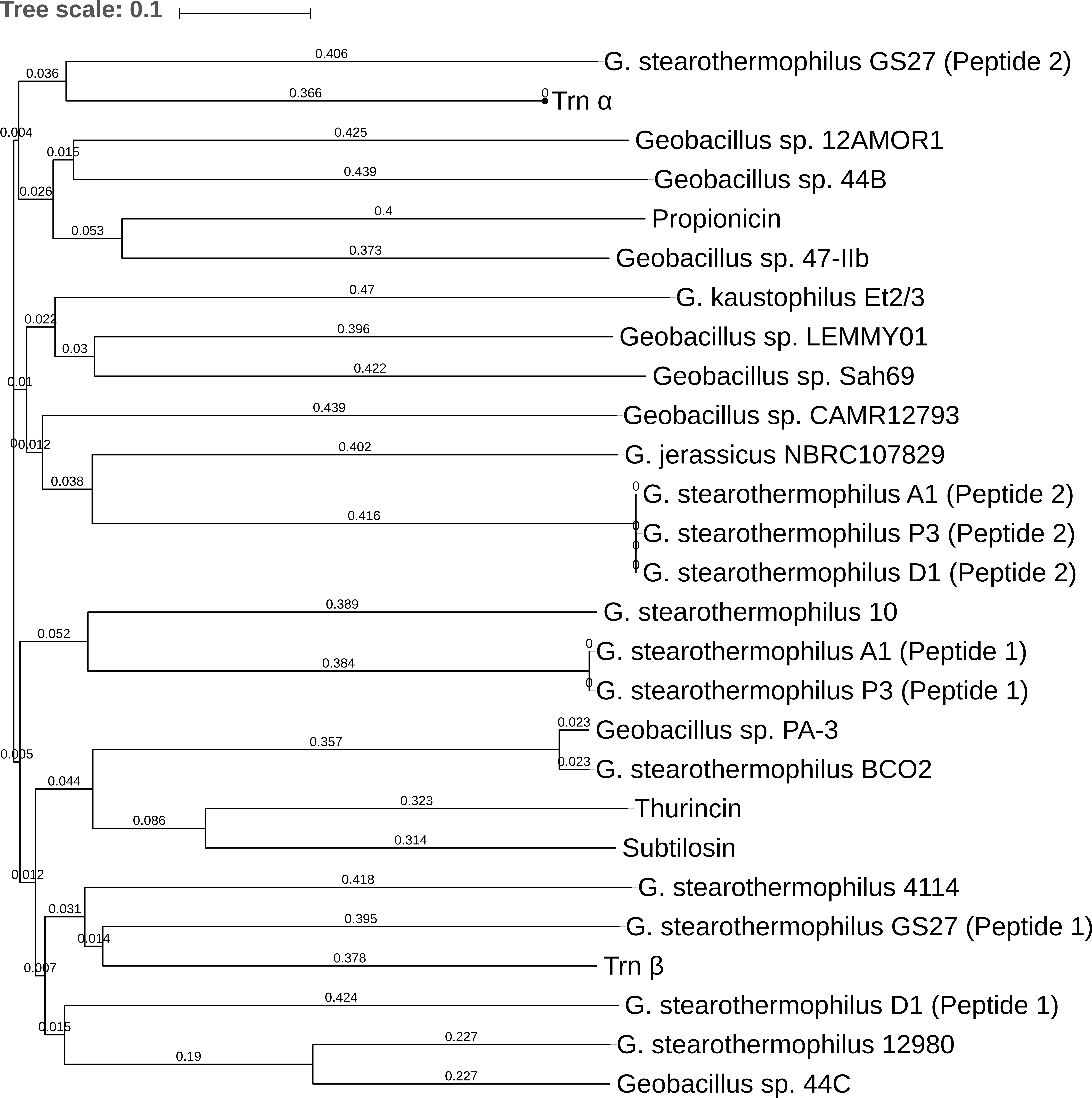

Supplement: Figure S2 — Phylogenetic arrangement of predicted sactibiotics. [file Image_2.JPEG]

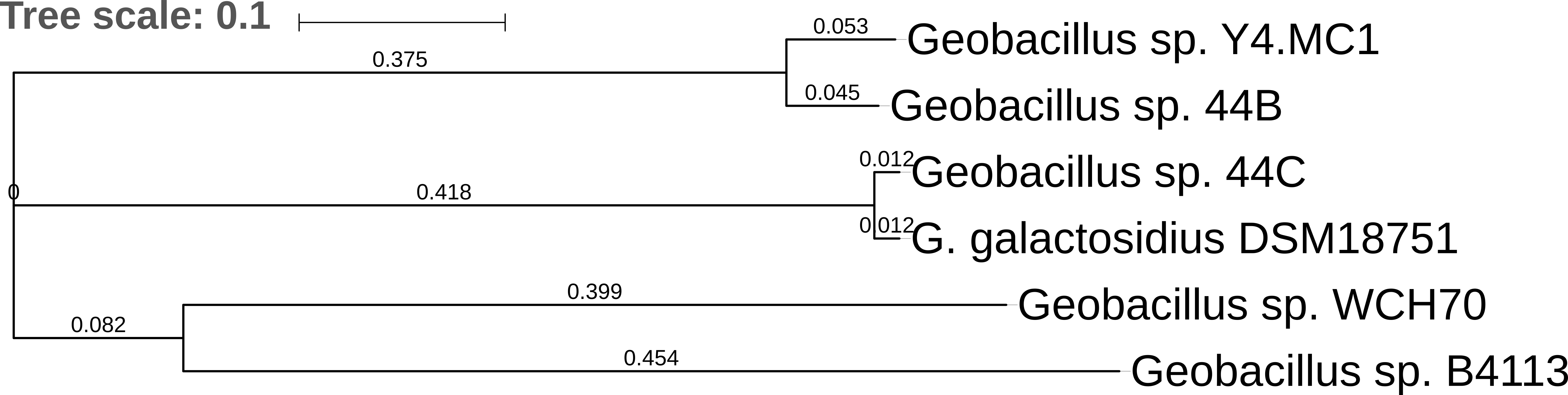

Supplement: Figure S3 — Phylogenetic arrangement of predicted LAPs. [file Image_3.JPEG]

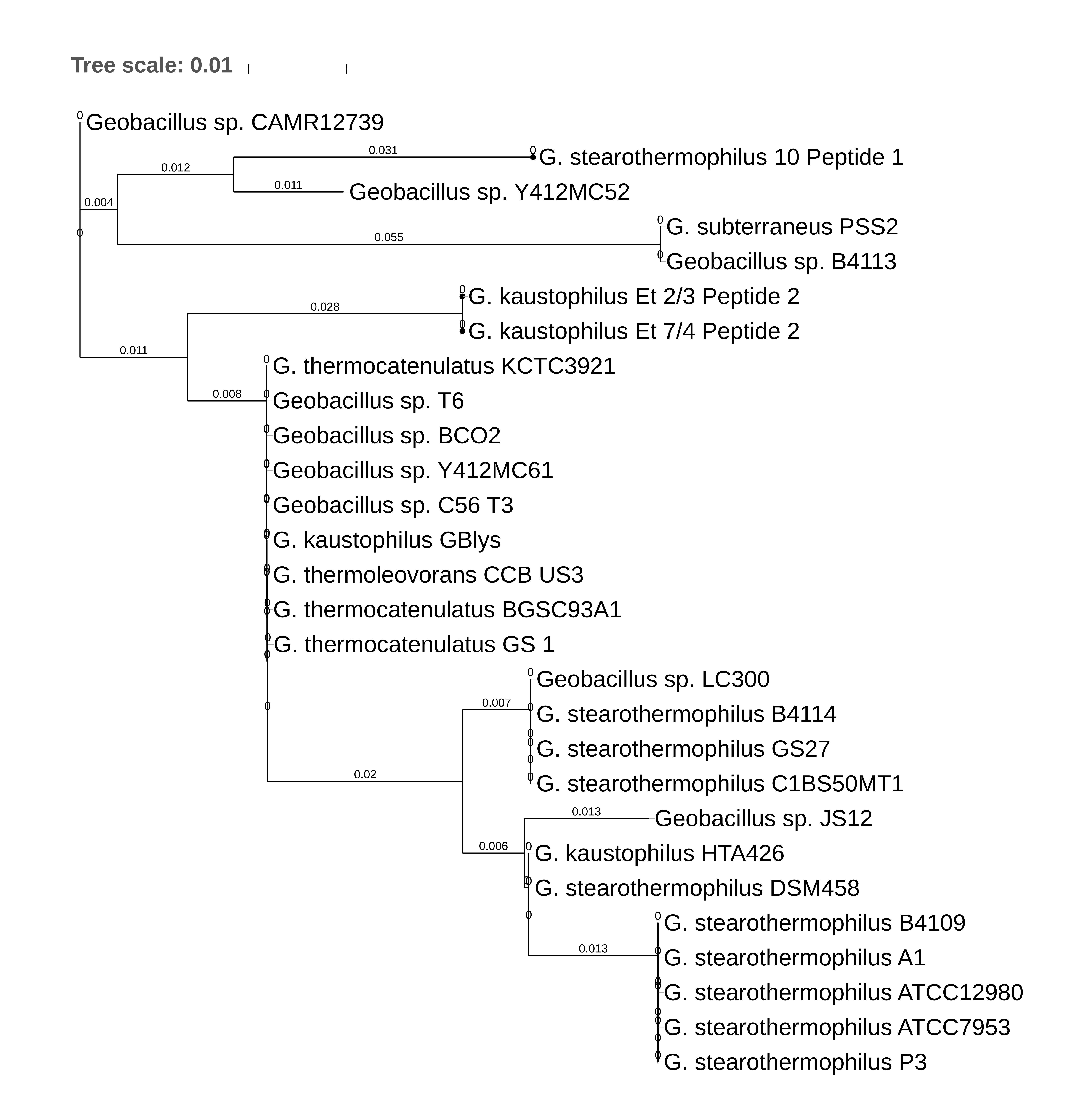

Supplement: Figure S4 — Phylogenetic arrangment of predicted circular bacteriocins. [file Image_4.JPEG]

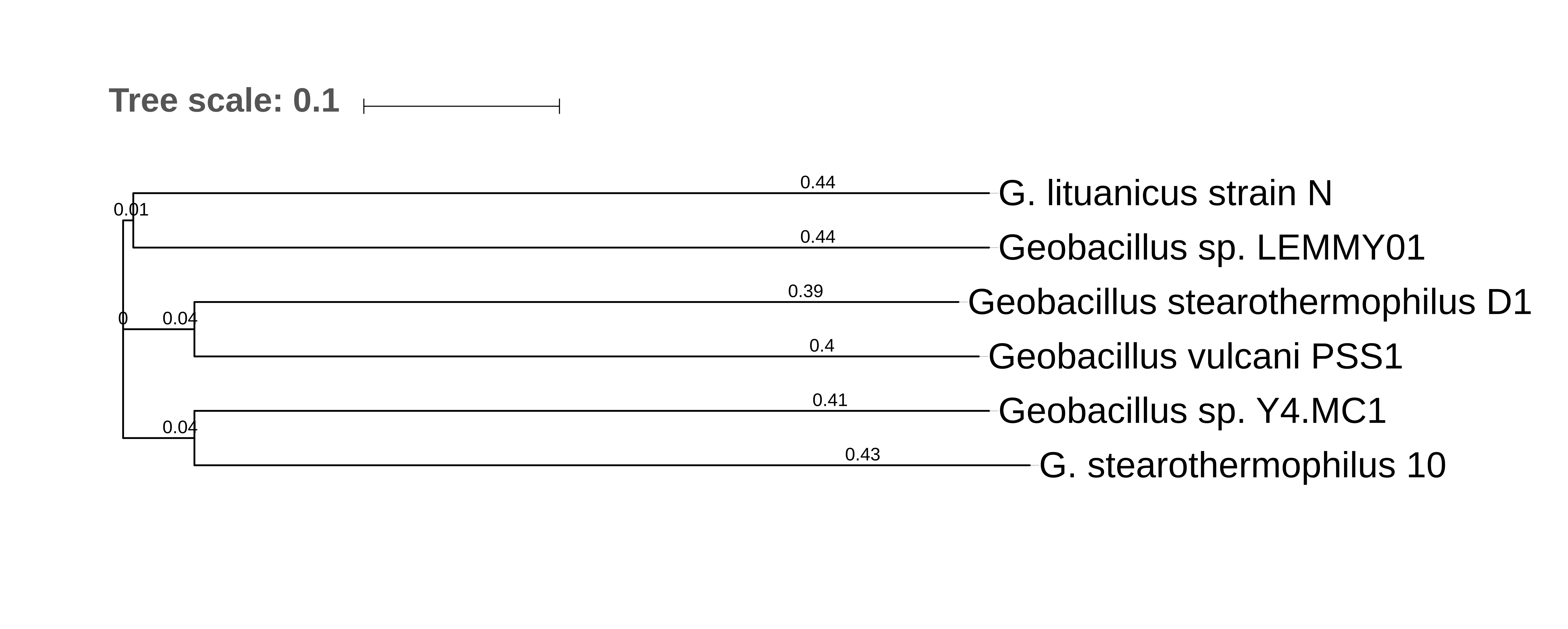

Supplement: Figure S5 — Phylogenetic arrangement of predicted class II bacteriocins. [file Image_5.JPEG]
